# The Influence of Socioeconomic Status on Racial/Ethnic Disparities among the ER/PR/HER2 Breast Cancer Subtypes

**DOI:** 10.1155/2015/813456

**Published:** 2015-08-03

**Authors:** Carol A. Parise, Vincent Caggiano

**Affiliations:** Sutter Institute for Medical Research, Sacramento, CA, USA

## Abstract

*Background*. The eight ER/PR/HER2 breast cancer subtypes vary widely in demographic and clinicopathologic characteristics and survival. This study assesses the contribution of SES to the risk of mortality for blacks, Hispanics, Asian/Pacific Islanders, and American Indians when compared with white women for each ER/PR/HER2 subtype. *Methods*. We identified 143,184 cases of first primary female invasive breast cancer from the California Cancer Registry between 2000 and 2012. The risk of mortality was computed for each race/ethnicity within each ER/PR/HER2 subtype. Models were adjusted for tumor grade, year of diagnosis, and age. SES was added to a second set of models. Analyses were conducted separately for each stage. *Results*. Race/ethnicity did not contribute to the risk of mortality for any subtype in stage 1 when adjusted for SES. In stages 2, 3, and 4, race/ethnicity was associated with risk of mortality and adjustment for SES changed the risk only in some subtypes. SES reduced the risk of mortality by over 45% for American Indians with stage 2 ER+/PR+/HER2− cancer, but it decreased the risk of mortality for blacks with stage 2 triple negative cancer by less than 4%. *Conclusions*. Racial/ethnic disparities do not exist in all ER/PR/HER2 subtypes and, in general, SES modestly alters these disparities.

## 1. Introduction

Disparities in the incidence and mortality of breast cancer among white, African American, Hispanic, Asian, and American Indian women have been well documented [1–5]. Survival differences have been attributed to a host of factors including demographic, clinicopathologic, genetic, treatment, lifestyle, and socioeconomic status (SES) [6–14].

The relationship between race/ethnicity and SES is complicated and it remains difficult to completely unravel their respective roles in breast cancer outcomes. This conundrum is evident from the conflicting results of studies investigating racial/ethnic disparities in cancer. Some have shown comparable outcomes after adjustment for sociodemographic factors if patients have equal access to healthcare [10, 15–19]. Others have shown that racial disparities persist even after adjusting for SES and despite equal access to healthcare [20–22]. Further, some studies have found that low SES, not race/ethnicity, was associated with poorer outcomes [23–25].

A factor that is seldom addressed in the research of race/ethnicity and SES in breast cancer disparities are the breast cancer tumor markers estrogen receptor (ER), progesterone receptor (PR), and human epidermal growth factor receptor 2 (HER2). These tumor markers are well known to clinicians, readily available, inexpensive, reproducible, reliable, and recorded in most tumor registries. Although these tumor markers have demonstrated wide variability in incidence and survival, most disparities research considers breast cancer as a single disease [4, 26].

O'Malley and colleagues explored the role of SES on racial/ethnic differences in breast cancer survival when adjusted for clinical variables including ER and PR status but not HER2 status and found that black women continued to have slight but significantly poorer survival when compared with white women [27]. This study prompts the question of whether racial disparities in breast cancer vary by ER/PR/HER2.

The objective of this study is to examine the contribution of SES to racial disparities in breast cancer mortality by assessing the risk of mortality for African American, Hispanic, Asian/Pacific Islanders, and American Indians within each of the ER/PR/HER2 subtypes.

## 2. Methods

The study included cases of first primary female invasive breast cancer diagnosed between January 1, 2000, and December 31, 2012, and reported to the California Cancer Registry (CCR) in December, 2013 (ICDO-3 sites C50.0–C50.9) [28]. Cases were reported to the Cancer Surveillance Section of the California Department of Public Health from hospitals and other facilities providing care or therapy to cancer patients residing in California [29]. Cases identified outside of California, only at autopsy, or only from death certificates were excluded.

Of the 245,701 cases, 102,517 were missing data for the cause of death, American Joint Commission on Cancer (AJCC) stage, age, ER/PR/HER2 subtype, tumor grade, tumor size, race/ethnicity, or socioeconomic status so that 143,184 cases had complete data available for all analyses.

### 2.1. Socioeconomic Status

SES was derived using data from the 2000 United States census for cases diagnosed from 2000 to 2005, whereas, for cases diagnosed from 2006 to 2012, data from the American Community Survey were used [30]. This SES variable is an index that uses education, employment characteristics, median household income, proportion of the population living 200% below the Federal Poverty Level, median rent, and median housing value of census tract of residence for case and denominator population. A principal component analysis was used to identify quintiles of SES ranging from 1 (the lowest) to 5 (the highest) [31]. This area based SES measure has been used in many studies utilizing cancer registry data [32–36].

### 2.2. ER/PR/HER2

The details of documentation of ER, PR, and HER2 along with age, stage at diagnosis, and tumor grade have been extensively described in our previous publications [26, 33, 36–38]. The eight subtypes are defined as ER+/PR+/HER2−, ER+/PR+/HER2+, ER+/PR−/HER2−, ER+/PR−/HER2+, ER−/PR+/HER2−, ER−/PR+/HER2+, ER−/PR−/HER2− (triple negative), and ER−/PR−/HER2+ (HER2-overexpressing).

### 2.3. Race/Ethnicity

Race/ethnicity was classified into 5 mutually exclusive categories: non-Hispanic white, African American/black, Hispanic, American Indian or Alaskan native, and Asian/Pacific Islander. Race/ethnicity was based on the information obtained from the medical record, which was derived from patient self-identification, assumptions based on personal appearance, or inferences based on the race/ethnicity of the parents, birthplace, surname, or maiden name. Hispanic ethnicity was based on the information from the medical record and computerized comparisons to the 1980 US census list of Hispanic surnames. Patients identified as Hispanic on the medical record or patients identified as white, black, or of unknown race with a Hispanic surname were classified as Hispanic.

### 2.4. Statistical Analysis

Kaplan-Meier survival analysis and the Log-Rank test were used to assess differences in survival among the ER/PR/HER2 subtypes. Cox Proportional Hazards modeling was used to compute the risk of mortality for each race/ethnicity when compared with white women for each ER/PR/HER2 subtype except the ER−/PR+/HER2− and ER−/PR+/HER2+ subtypes. These subtypes lacked sufficient cases.

The first models adjusted race/ethnicity for tumor grade, year of diagnosis, and age. Tumor size was not included because of its high correlation with AJCC stage. SES was added to the second set of models. Analyses were conducted separately for each AJCC stage because of the differences in prognosis of patients diagnosed in different stages.

Hazard ratios (HRs) and 95% confidence intervals (CI) were computed for all models and represented the risk of mortality for each race/ethnicity relative to white women with the same stage and ER/PR/HER2 subtype. The HRs were only interpreted when the Wald *X* ^2^ for race/ethnicity was statistically significant (*P* < 0.05). All analyses were conducted using SPSS 21.0 [39].

This research study involved analysis of existing data from the CCR without subject identifiers or intervention. Therefore, the study was categorized as exempt from institutional review board oversight.

## 3. Results

Table 1 displays the demographic and tumor characteristics of each of the race/ethnicities included in the study. Median follow-up time was 54 months with a maximum of 155 months. The ER+/PR+/HER2− subtype was the predominant subtype and represented 58.1% of all cases followed by the triple negative subtype at 12.8%. The ER-positive subtypes represented almost 80% of all cases, but there was wide variation by race/ethnicity.

Blacks were the only race/ethnicity where fewer than 50% of cases were the ER+/PR+/HER2− subtype, but they had the highest percent of triple negative cases. Asian/Pacific Islanders were least likely to have the triple negative subtype but made up a higher proportion of the ER−/PR−/HER2+ subtype than any other race/ethnicity.

Whites and Asian/Pacific Islanders were more likely to be in the highest SES and diagnosed in stage 1 and blacks and Hispanics in the lowest SES. American Indians and blacks were more likely to be diagnosed in stage 4.

**Figure 1:**
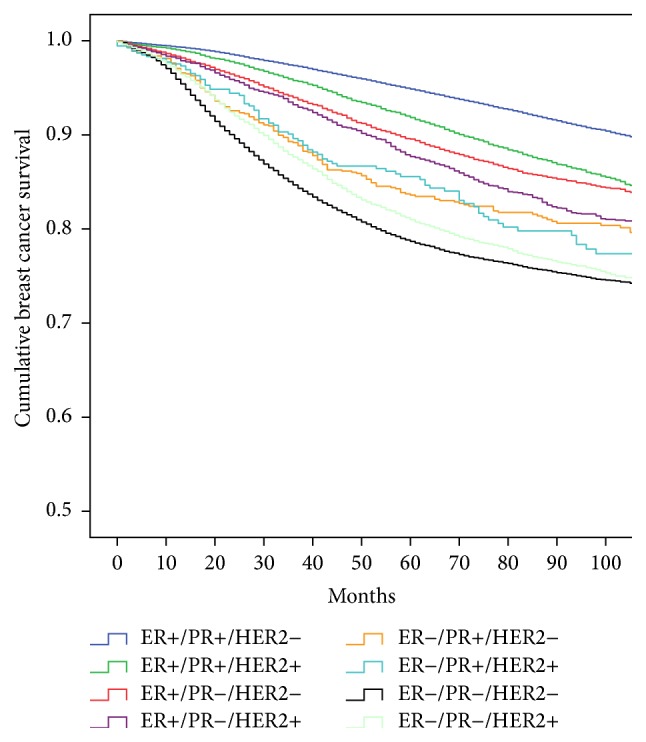
Unadjusted Kaplan-Meier breast cancer specific survival of the eight ER/PR/HER2 subtypes in 143,184 cases from the California Cancer Registry 2000–2012.

Table 2 and Figure 1 demonstrate that all of the ER-positive subtypes had better 5-year survival than the ER-negative subtypes, and the ER+/PR+/HER2− subtype had the best overall survival, statistically significantly better than all other subtypes (*P* < 0.001). The triple negative subtype had the worst overall survival followed by the ER−/PR−/HER2+ subtype.

The heterogeneity of the HER2-positive subtypes and the influence of ER-positivity were evident with the ER+/PR+/HER2+ subtype having 92.0% survival contrasting with the ER−/PR−/HER2+ subtype having an 81.1% survival. The importance of PR status was noted when comparing the subtypes that differ only by its presence or absence.

Cox Proportional Hazards models revealed that the contribution of SES to the survival disparities in race/ethnicity varied by ER/PR/HER2 and stage at diagnosis. For several stages and subtypes, the Wald *X* ^2^ was not statistically significant, indicating that race/ethnicity did not contribute to the risk of breast cancer specific mortality.

### 3.1. Stage 1

When unadjusted for SES, blacks with the ER+/PR+/HER2+ subtype had over 2 times the risk of mortality as whites (HR = 2.22; 95% CI = 1.31–3.77). However, when SES was included in the model in stage 1, race/ethnicity did not contribute to the risk of mortality for any subtype.

### 3.2. Stage 2

Blacks had an increased risk mortality for the ER+/PR+/HER2− (HR = 1.51; 95% CI = 1.28–1.78), ER+/PR+/HER2+ (HR = 1.79; 95% CI = 1.35–2.37), and triple negative subtypes (HR = 1.36; 95% CI = 1.18–1.56) unadjusted for SES. The models with SES reduced the risk for the ER+/PR+/HER2− (HR = 1.32; 95% CI = 1.12–1.56), ER+/PR+/HER2+ (HR = 1.58; 95% CI = 1.18–2.11), and triple negative subtypes (HR = 1.32; 95% CI = 1.14–1.53). American Indians had an increased HR for the ER+/PR+/HER2− subtype without SES (HR = 2.20; 95% CI = 1.38–3.51) which was reduced with the inclusion of SES (HR = 1.91; 95% CI = 1.20–3.05). Hispanics had a reduced risk of death in the ER+/PR+/HER2− but only in the presence of SES (HR = 0.84; 95% CI = 0.73–0.95). However, Hispanics with the ER+/PR+/HER2+ subtype had worse survival than whites in the same stage and subtype (HR = 1.28; 95% CI = 1.05–1.57), but this risk was not statistically significant in the presence of SES.

### 3.3. Stage 3

Race/ethnicity was statistically significantly associated with risk of mortality only for the ER+/PR−/HER2− and triple negative subtypes. Blacks with ER+/PR−/HER2− subtype (HR = 1.56; 95% CI = 1.13–2.17) and triple negative subtypes (HR = 1.36; 95% CI = 1.15–1.61) had worse survival than whites with the same subtype and stage. Inclusion of SES reduced this risk by 7% for the ER+/PR−/HER2− subtype (HR = 1.49; 95% CI = 1.07–2.08) and less than 1% for the triple negative subtype (HR = 1.33; 95% CI = 1.12–1.59). Asian/Pacific Islanders with the ER+/PR−/HER2− subtype had a 33% reduction in risk of death over whites (HR = 0.66; 95% CI = 0.45–0.98) but only when SES was included in the model.

### 3.4. Stage 4

African Americans with the ER+/PR+/HER2− (HR = 1.46; 95% CI = 1.17–1.81) and triple negative subtypes (HR = 1.42; 95% CI = 1.10–1.84) had an increased risk of death over whites in stage 4. Adjusting for SES reduced this risk by 9% in the ER+/PR+/HER2− subtype (HR = 1.37; 95% CI = 1.10–1.72), but, with inclusion of SES, race/ethnicity was no longer a statistically significant risk factor for survival for the triple negative subtype.

Asian/Pacific Islanders with the ER+/PR+/HER2− subtype had a 25% lower risk of mortality (HR = 0.75; 95% CI = 0.59–0.96) which was reduced by only 1% when adjusted for SES (HR = 0.74; 95% CI = 0.58–0.94).

## 4. Discussion

This study has shown that the contribution of SES to racial/ethnic disparities varies considerably for each of the ER/PR/HER2 subtypes.

It is generally acknowledged that breast cancer is a heterogeneous disease based on gene expression patterns with different outcomes, responses to treatment, and racial/ethnic distribution [26, 37, 40–43] but with few exceptions [44–50], most disparities in health care investigations have considered breast cancer as a single disease. Many investigators convert these ER/PR/HER2 subtypes into molecular surrogate subtypes resulting in Luminal A (ER- and/or PR-positive, HER2-negative), Luminal B (ER- and/or PR-positive, HER2-positive, basal or triple negative), and HER2-overexpressing (ER- and PR-negative, HER2-positive). However, the exact immunohistochemical equivalent of Luminal B remains controversial or requires additional testing or use of tumor grade [12, 51–56]. Additionally, many studies define the term hormone receptor positive to be “ER- and/or PR-positive” which may mask the heterogeneity by combining ER-positive with PR-negative, or vice versa, ER-negative with PR-positive [51, 52]. However defined, these subtypes have different outcomes, responses to treatment, and racial/ethnic distribution [26, 40–43, 45].

To our knowledge, this is the first study to address the contribution of SES to racial disparities in breast cancer within stage of disease and the individual ER/PR/HER2 subtypes.

In stage 1, there are no racial/ethnic disparities for any subtype when controlling for SES which suggests that innate biological differences among the ethnicities, at least for this stage, appear unlikely consistent with previous research [17, 18, 23, 57]. In contrast, others found a black/white disparity even after adjusting for SES and other variables [22, 27].

In stages 2, 3, and 4, race/ethnicity is associated with risk of mortality, and adjustment with SES changed this risk only in some subtypes. The most extreme case is where SES reduced the risk of mortality by over 45% for American Indians with the ER+/PR+/HER2− subtype in stage 2. However, for stage 2 triple negative cases, SES decreased the risk of mortality for blacks by less than 4%. Asian/Pacific Islanders have traditionally been found to have equal or better survival than whites [5, 58, 59]. This advantage is not as apparent when stratified by stage and subtype.

These results provide further evidence for the heterogeneity of breast cancer and emphasize the use of the eight ER/PR/HER2 subtypes. The variation in racial disparities is particularly evident in the higher stages of disease, and, as stated in our prior research, it is unknown if tumor or host factors play a role in advanced stages of disease or if there is an element of racial/ethnic discrimination in receipt of more aggressive cancer treatments [57].

Our findings might suggest that there is progress in the elimination of disparities in breast cancer survival. However, our descriptive data and previous research project a completely different picture [26]. In most instances, white women present with favorable tumor and demographic conditions and black women with unfavorable conditions. White women are more likely to present in stage 1 with small, grade 1 ER+/PR+/HER2− subtype tumors and reside in the highest SES strata. Conversely, black women have the lowest proportion of ER+/PR+/HER2− cases and present at later stages with higher grade tumors and are in the lowest SES strata. Black women are also more likely to present with ER-negative breast cancer, especially the triple negative subtype. Although younger age or premenopausal status is an important risk factor for the triple negative subtype in white women, African ancestry may be more important in black women [45, 60–62]. These differences continue to impact racial/ethnic disparities, especially the black/white disparity, and it appears that little has changed over time [63, 64].

The limitations of population-based cancer registry investigations, including missing data, especially ER, PR, and HER2, lack of central pathology review, and comorbid conditions have been described in our prior publications [32, 33, 38, 57]. The determination of race/ethnicity can be problematic, arbitrary, and subject to error [57, 65]. Accurate and precise treatment information was not available from the registry. Although it has been suggested that suboptimal use of adjuvant treatments may explain differences in outcomes [6, 66–70], others have reported little or no racial/ethnic differences with regard to chemotherapy administration [71–73]. The CCR does not have the ability to obtain individual level SES so our measure of SES was at the neighborhood level rather than the individual level. However, this measure of SES has been used in many studies that utilize cancer registry data [33, 34, 38, 74, 75] and many have commented on the usefulness of composite SES measures [24, 76–80]. Nevertheless, nondifferential misclassification of cases by SES was possible, which would bias the results toward the null.

Additionally, the registry has no information about reproductive history and lifestyle risk factors such as nulliparity, multiparity, breast feeding, diet, body fat distribution, use of alcohol, oral contraceptives, or hormone replacement treatments that may determine the type of breast cancer and ultimately impact survival [81–89].

The strengths of this study include the large number of cases reported to the statewide cancer registry from an ethnically diverse population, maximum follow-up of almost 13 years, and use of the individual ER/PR/HER2 subtypes.

In conclusion, we have shown that, in the state of California, racial/ethnic disparities in breast cancer survival do not exist for all eight ER/PR/HER2 subtypes. Further, the contribution of SES to racial/ethnic disparities varies by ER/PR/HER2 subtype and stage at diagnosis, and, in most instances, it is quite modest. Continued research is warranted in genetic, societal, and lifestyle factors which are associated with poor breast cancer survival.

## Figures and Tables

**Table 1:** Demographic and clinicopathologic characteristics of 143,184 AJCC stages 1–4 of first primary female invasive breast cancer from the California Cancer Registry 2000–2012^*∗*^.

	White (*n* = 93,325)	Black (*n* = 8,718)	Hispanic (*n* = 24,078)	Asian/Pacific Islander (*n* = 16,476)	American Indian (*n* = 587)	Total *N* = 143,184
Mean age in years ± SD	61.49 ± 13.47	57.72 ± 13.51	55.13 ± 13.35	55.87 ± 12.93	57.50 ± 12.44	59.53 ± 13.67
Age						
<45	12.1%	19.4%	25.5%	22.6%	16.5%	22,944
46–69	58.9%	59.9%	58.4%	61.1%	66.1%	84,704
70+	29.0%	20.7%	16.0%	16.2%	17.4%	35,536

AJCC stage						
Stage 1	50.7%	37.1%	38.3%	45.0%	43.8%	67,435
Stage 2	37.1%	43.4%	42.8%	41.6%	38.3%	55,782
Stage 3	9.5%	14.5%	15.3%	10.6%	13.6%	15,664
Stage 4	2.7%	5.1%	3.6%	2.8%	4.3%	4,303

ER/PR/HER2 subtype						
ER+/PR+/HER2−	61.3%	44.2%	52.2%	56.1%	56.9%	83,169
ER+/PR+/HER2+	8.7%	8.9%	10.3%	11.3%	9.5%	13,293
ER+/PR−/HER2−	9.7%	10.1%	8.7%	8.0%	9.7%	13,363
ER+/PR−/HER2+	3.0%	3.3%	3.4%	3.6%	3.6%	4,535
ER−/PR+/HER2−	0.7%	1.1%	1.0%	0.8%	0.7%	1,131
ER−/PR+/HER2+	0.3%	0.6%	0.6%	0.4%	0.2%	539
ER−/PR−/HER2−	11.2%	24.5%	15.9%	11.0%	14.0%	18,299
ER−/PR−/HER2+	5.2%	7.3%	7.9%	8.8%	5.5%	8,855

Socioeconomic status (SES)						
SES1-low	6.6%	25.4%	28.0%	7.2%	18.4%	16,424
SES2	13.8%	24.9%	24.4%	14.3%	25.7%	23,383
SES3	20.2%	21.8%	20.1%	19.3%	26.1%	28,953
SES4	26.1%	17.6%	16.2%	26.9%	18.7%	34,332
SES5-high	33.3%	10.3%	11.4%	32.3%	11.1%	40,092

Tumor grade						
Well differentiated; grade I (low)	25.6%	14.5%	16.9%	18.3%	20.3%	32,367
Moderately differentiated; grade II (low)	43.7%	35.1%	40.2%	43.0%	42.6%	60,820
Poorly differentiated; grade III (high)	29.5%	48.4%	41.1%	37.3%	36.1%	47,969
Undifferentiated; grade IV (high)	1.3%	2.0%	1.8%	1.3%	1.0%	2,028

Tumor size (mm)						
<1–4.99	6.3%	4.8%	5.0%	6.7%	5.6%	8,650
5.00–9.99	18.7%	12.0%	12.6%	14.5%	14.3%	23,968
10.00–19.99	38.6%	33.0%	34.2%	36.1%	35.8%	53,306
20.00–49.99	28.7%	36.9%	36.6%	34.0%	32.9%	44,623
50.00+	7.7%	13.3%	11.6%	8.6%	11.4%	12,637

*∗* includes cases with complete data for ER/PR/HER2, age, AJCC stage, tumor grade, tumor size, race/ethnicity, and socioeconomic status.

**Table 2:** Five-year survival of the eight ER/PR/HER2 subtypes^*∗*^.

ER/PR/HER2	*n*	%	5-year survival	95% CI
ER+/PR+/HER2−	83,169	58.09%	94.95%	(94.86%, 95.12%)
ER+/PR+/HER2+	13,293	9.28%	92.00%	(91.73%, 92.52%)
ER+/PR−/HER2−	13,363	9.33%	89.58%	(89.27%, 90.17%)
ER+/PR−/HER2+	4,535	3.17%	87.87%	(87.31%, 88.96%)
ER−/PR+/HER2−	1,131	0.79%	83.68%	(82.46%, 86.08%)
ER−/PR+/HER2+	539	0.38%	85.53%	(83.85%, 88.83%)
ER−/PR−/HER2−	18,299	12.78%	78.78%	(78.44%, 79.45%)
ER−/PR−/HER2+	8,855	6.18%	81.11%	(80.65%, 82.02%)

Total	143,184			

^*∗*^Survival for all subtypes statistically significantly worse (Log-Rank test *P* < 0.001) than the ER+/PR+/HER2− subtype.

## Disclaimer

The collection of cancer incidence data used in this study was supported by the California Department of Public Health as part of the statewide cancer reporting program mandated by California Health and Safety Code Section 103885; the National Cancer Institute's Surveillance, Epidemiology and End Results Program under Contract HHSN261201000140C awarded to the Cancer Prevention Institute of California, Contract HHSN261201000035C awarded to the University of Southern California, and Contract HHSN261201000034C awarded to the Public Health Institute; and the Centers for Disease Control and Prevention's National Program of Cancer Registries, under Agreement U58DP003862-01 awarded to the California Department of Public Health. The ideas and opinions expressed herein are those of the authors and endorsement by the State of California, Department of Public Health the National Cancer Institute, and the Centers for Disease Control and Prevention or their Contractors and Subcontractors is not intended nor should be inferred.

## References

[1] Ries L., Eisner M., Kosary M. (2003). *SEER Cancer Statistics Review, 1975–2000*.

[2] Silber J. H., Rosenbaum P. R., Clark A. S. (2013). Characteristics associated with differences in survival among black and white women with breast cancer. *Journal of the American Medical Association*.

[3] Tannenbaum S. L., Koru-Sengul T., Miao F., Byrne M. M. (2013). Disparities in survival after female breast cancer diagnosis: a population-based study. *Cancer Causes and Control*.

[4] Parise C., Caggiano V. (2014). Disparities in the risk of the ER/PR/HER2 breast cancer subtypes among Asian Americans in California. *Cancer Epidemiology*.

[5] Iqbal J., Ginsburg O., Rochon P. A., Sun P., Narod S. A. (2015). Differences in breast cancer stage at diagnosis and cancer-specific survival by race and ethnicity in the United States. *Journal of the American Medical Association*.

[6] Shavers V. L., Harlan L. C., Stevens J. L. (2003). Racial/ethnic variation in clinical presentation, treatment, and survival among breast cancer patients under age 35. *Cancer*.

[7] Chlebowski R. T., Chen Z., Anderson G. L. (2005). Ethnicity and breast cancer: factors influencing differences in incidence and outcome. *Journal of the National Cancer Institute*.

[8] Hershman D., McBride R., Jacobson J. S. (2005). Racial disparities in treatment and survival among women with early-stage breast cancer. *Journal of Clinical Oncology*.

[9] Menashe I., Anderson W. F., Jatoi I., Rosenberg P. S. (2009). Underlying causes of the black-white racial disparity in breast cancer mortality: a population-based analysis. *Journal of the National Cancer Institute*.

[10] Vona-Davis L., Rose D. P. (2009). The influence of socioeconomic disparities on breast cancer tumor biology and prognosis: a review. *Journal of Women's Health*.

[11] Deshpande A. D., Jeffe D. B., Gnerlich J., Iqbal A. Z., Thummalakunta A., Margenthaler J. A. (2009). Racial disparities in breast cancer survival: an analysis by age and stage. *Journal of Surgical Research*.

[12] Bhargava A., Du X. L. (2009). Racial and socioeconomic disparities in adjuvant chemotherapy for older women with lymph node-positive, operable breast cancer. *Cancer*.

[13] Brawley O. W. (2013). Health disparities in breast cancer. *Obstetrics and Gynecology Clinics of North America*.

[14] Wheeler S. B., Reeder-Hayes K. E., Carey L. A. (2013). Disparities in breast cancer treatment and outcomes: biological, social, and health system determinants and opportunities for research. *Oncologist*.

[15] Roach M., Cirrincione C., Budman D. (1997). Race and survival from breast cancer: based on Cancer and Leukemia Group B trial 8541. *Cancer Journal from Scientific American*.

[16] Curtis E., Quale C., Haggstrom D., Smith-Bindman R. (2008). Racial and ethnic differences in breast cancer survival: how much is explained by screening, tumor severity, biology, treatment, comorbidities, and demographics?. *Cancer*.

[17] Du X. L., Fang S., Meyer T. E. (2008). Impact of treatment and socioeconomic status on racial disparities in survival among older women with breast cancer. *American Journal of Clinical Oncology: Cancer Clinical Trials*.

[18] Chu Q. D., Smith M. H., Williams M. (2009). Race/ethnicity has no effect on outcome for breast cancer patients treated at an academic center with a public hospital. *Cancer Epidemiology Biomarkers and Prevention*.

[19] Komenaka I. K., Martinez M. E., Pennington R. E. (2010). Race and ethnicity and breast cancer outcomes in an underinsured population. *Journal of the National Cancer Institute*.

[20] Newman L. A., Griffith K. A., Jatoi I., Simon M. S., Crowe J. P., Colditz G. A. (2006). Meta-analysis of survival in African American and white American patients with breast cancer: ethnicity compared with socioeconomic status. *Journal of Clinical Oncology*.

[21] Albain K. S., Unger J. M., Crowley J. J., Coltman C. A., Hershman D. L. (2009). Racial disparities in cancer survival among randomized clinical trials patients of the southwest oncology group. *Journal of the National Cancer Institute*.

[22] Keegan T. H., Kurian A. W., Gali K. (2015). Racial/ethnic and socioeconomic differences in short-term breast cancer survival among women in an integrated health system. *American Journal of Public Health*.

[23] Bradley C. J., Given C. W., Roberts C. (2002). Race, socioeconomic status, and breast cancer treatment and survival. *Journal of the National Cancer Institute*.

[24] Byers T. E., Wolf H. J., Bauer K. R. (2008). The impact of socioeconomic status on survival after cancer in the United States: findings from the National Program of Cancer Registries patterns of care study. *Cancer*.

[25] Sprague B. L., Trentham-Dietz A., Gangnon R. E. (2011). Socioeconomic status and survival after an invasive breast cancer diagnosis. *Cancer*.

[26] Parise C. A., Bauer K. R., Brown M. M., Caggiano V. (2009). Breast cancer subtypes as defined by the estrogen receptor (ER), progesterone receptor (PR), and the human epidermal growth factor receptor 2 (HER2) among women with invasive breast cancer in California, 1999–2004. *The Breast Journal*.

[27] O'Malley C. D., Le G. M., Glaser S. L., Shema S. J., West D. W. (2003). Socioeconomic status and breast carcinoma survival in four racial/ethnic groups: a population-based study. *Cancer*.

[28] Fritz A. G. (2000). *International Classification of Diseases for Oncology: ICD-O*.

[29] California Department of Public; Cancer Surveillance and Research Branch (2008). Cancer reporting in California: abstracting and coding procedures for hospitals. *California Cancer Reporting System Standards*.

[30] https://www.census.gov/acs/www/data_documentation/summary_file/.

[31] Yost K., Perkins C., Cohen R., Morris C., Wright W. (2001). Socioeconomic status and breast cancer incidence in California for different race/ethnic groups. *Cancer Causes and Control*.

[32] Bauer K. R., Brown M., Creech C., Schlag N. C., Caggiano V. (2007). Data quality assessment of HER2 in the Sacramento region of the California cancer registry. *Journal of Registry Management*.

[33] Brown M., Tsodikov A., Bauer K. R., Parise C. A., Caggiano V. (2008). The role of human epidermal growth factor receptor 2 in the survival of women with estrogen and progesterone receptor-negative, invasive breast cancer: the California Cancer Registry, 1999–2004. *Cancer*.

[34] Clarke C. A., Glaser S. L., Keegan T. H. M., Stroup A. (2005). Neighborhood socioeconomic status and Hodgkin's lymphoma incidence in California. *Cancer Epidemiology Biomarkers and Prevention*.

[35] Parikh-Patel A., Mills P. K., Jain R. V. (2006). Breast cancer survival among South Asian women in California (United States). *Cancer Causes and Control*.

[36] Parise C. A., Bauer K. R., Caggiano V. (2012). Disparities in receipt of adjuvant radiation therapy after breast-conserving surgery among the cancer-reporting regions of California. *Cancer*.

[37] Bauer K., Parise C., Caggiano V. (2010). Use of ER/PR/HER2 subtypes in conjunction with the 2007 St Gallen Consensus Statement for early breast cancer. *BMC Cancer*.

[38] Bauer K. R., Brown M., Cress R. D., Parise C. A., Caggiano V. (2007). Descriptive analysis of estrogen receptor (ER)-negative, progesterone receptor (PR)-negative, and HER2-negative invasive breast cancer, the so-called triple-negative phenotype: a population-based study from the California Cancer Registry. *Cancer*.

[39] IBM (2012). *IBM SPSS Statistics for Windows*.

[40] Perou C. M., Sørile T., Eisen M. B. (2000). Molecular portraits of human breast tumours. *Nature*.

[41] Sørlie T., Tibshirani R., Parker J. (2003). Repeated observation of breast tumor subtypes in independent gene expression data sets. *Proceedings of the National Academy of Sciences of the United States of America*.

[42] Carey L. A., Perou C. M., Livasy C. A. (2006). Race, breast cancer subtypes, and survival in the Carolina Breast Cancer Study. *Journal of the American Medical Association*.

[43] Cheang M. C. U., Voduc K. D., Tu D. (2012). Responsiveness of intrinsic subtypes to adjuvant anthracycline substitution in the NCIC.CTG MA.5 randomized trial. *Clinical Cancer Research*.

[44] Dunnwald L. K., Rossing M. A., Li C. I. (2007). Hormone receptor status, tumor characteristics, and prognosis: a prospective cohort of breast cancer patients. *Breast Cancer Research*.

[45] Parise C. A., Bauer K. R., Caggiano V. (2010). Variation in breast cancer subtypes with age and race/ethnicity. *Critical Reviews in Oncology/Hematology*.

[46] Kurian A. W., Fish K., Shema S. J., Clarke C. A. (2010). Lifetime risks of specific breast cancer subtypes among women in four racial/ethnic groups. *Breast Cancer Research*.

[47] Dunn B. K., Agurs-Collins T., Browne D., Lubet R., Johnson K. A. (2010). Health disparities in breast cancer: biology meets socioeconomic status. *Breast Cancer Research and Treatment*.

[48] Clarke C. A., Keegan T. H. M., Yang J. (2012). Age-specific incidence of breast cancer subtypes: understanding the black-white crossover. *Journal of the National Cancer Institute*.

[49] Prat A., Carey L. A., Adamo B. (2014). Molecular features and survival outcomes of the intrinsic subtypes within HER2-positive breast cancer. *Journal of the National Cancer Institute*.

[50] Sineshaw H. M., Gaudet M., Ward E. M. (2014). Association of race/ethnicity, socioeconomic status, and breast cancer subtypes in the National Cancer Data Base (2010-2011). *Breast Cancer Research and Treatment*.

[51] Howlader N., Altekruse S. F., Li C. I. (2014). US incidence of breast cancer subtypes defined by joint hormone receptor and HER2 status. *Journal of the National Cancer Institute*.

[52] Anderson W. F., Rosenberg P. S., Katki H. A. (2014). Tracking and evaluating molecular tumor markers with cancer registry data: HER2 and breast cancer. *Journal of the National Cancer Institute*.

[53] Maisonneuve P., Disalvatore D., Rotmensz N. (2014). Proposed new clinicopathological surrogate definitions of luminal A and luminal B (HER2-negative) intrinsic breast cancer subtypes. *Breast Cancer Research*.

[54] Goldhirsch A., Coates A. S., Gelber R. D., Glick J. H., Thürlimann B., Senn H.-J. (2006). First—select the target: better choice of adjuvant treatments for breast cancer patients. *Annals of Oncology*.

[55] Brouckaert O., Schoneveld A., Truyers C. (2013). Breast cancer phenotype, nodal status and palpability may be useful in the detection of overdiagnosed screening-detected breast cancers. *Annals of Oncology*.

[56] Engstrøm M. J., Opdahl S., Hagen A. I. (2013). Molecular subtypes, histopathological grade and survival in a historic cohort of breast cancer patients. *Breast cancer research and treatment*.

[57] Parise C. A., Caggiano V. (2013). Disparities in race/ethnicity and socioeconomic status: risk of mortality of breast cancer patients in the California Cancer Registry, 2000–2010. *BMC Cancer*.

[58] Wu A. H., Gomez S. L., Vigen C. (2013). The California Breast Cancer Survivorship Consortium (CBCSC): prognostic factors associated with racial/ethnic differences in breast cancer survival. *Cancer Causes and Control*.

[59] Trinh Q. D., Nguyen P. L., Leow J. J. (2015). Cancer-specific mortality of Asian Americans diagnosed with cancer: a nationwide population-based assessment. *Journal of the National Cancer Institute*.

[60] Stead L. A., Lash T. L., Sobieraj J. E. (2009). Triple-negative breast cancers are increased in black women regardless of age or body mass index. *Breast Cancer Research*.

[61] Amirikia K. C., Mills P., Bush J., Newman L. A. (2011). Higher population-based incidence rates of triple-negative breast cancer among young African-American women: implications for breast cancer screening recommendations. *Cancer*.

[62] Newman L. A. (2015). Disparities in breast cancer and african ancestry: a global perspective. *Breast Journal*.

[63] Aizer A. A., Wilhite T. J., Chen M.-H. (2014). Lack of reduction in racial disparities in cancer-specific mortality over a 20-year period. *Cancer*.

[64] Hunt B. R., Whitman S., Hurlbert M. S. (2014). Increasing black: white disparities in breast cancer mortality in the 50 largest cities in the United States. *Cancer Epidemiology*.

[65] Gomez S. L., Glaser S. L. (2006). Misclassification of race/ethnicity in a population-based cancer registry (United States). *Cancer Causes and Control*.

[66] Dragun A. E., Huang B., Tucker T. C., Spanos W. J. (2011). Disparities in the application of adjuvant radiotherapy after breast-conserving surgery for early stage breast cancer. *Cancer*.

[67] Bickell N. A., Wang J. J., Oluwole S. (2006). Missed opportunities: racial disparities in adjuvant breast cancer treatment. *Journal of Clinical Oncology*.

[68] Lund M. J., Brawley O. P., Ward K. C., Young J. L., Gabram S. S. G., Eley J. W. (2008). Parity and disparity in first course treatment of invasive breast cancer. *Breast Cancer Research and Treatment*.

[69] Li C. I., Daling J. R., Malone K. E. (2003). Incidence of invasive breast cancer by hormone receptor status from 1992 to 1998. *Journal of Clinical Oncology*.

[70] van Ravesteyn N. T., Schechter C. B., Near A. M. (2011). Race-specific impact of natural history, mammography screening, and adjuvant treatment on breast cancer mortality rates in the United States. *Cancer Epidemiology Biomarkers and Prevention*.

[71] Muss H. B., Hunter C. P., Wesley M. (1992). Treatment plans for black and white women with stage II node-positive breast cancer: the National Cancer Institute Black/White Cancer Survival Study Experience. *Cancer*.

[72] Harlan L. C., Abrams J., Warren J. L., Clegg L., Stevens J., Ballard-Barbash R. (2002). Adjuvant therapy for breast cancer: practice patterns of community physicians. *Journal of Clinical Oncology*.

[73] Griggs J. J. (2012). Role of nonclinical factors in the receipt of high-quality systemic adjuvant breast cancer treatment. *Journal of Clinical Oncology*.

[74] Parikh-Patel A., Bates J. H., Campleman S. (2006). Colorectal cancer stage at diagnosis by socioeconomic and urban/rural status in California, 1988–2000. *Cancer*.

[75] Zell J. A., Rhee J. M., Ziogas A., Lipkin S. M., Anton-Culver H. (2007). Race, socioeconomic status, treatment, and survival time among pancreatic cancer cases in California. *Cancer Epidemiology Biomarkers and Prevention*.

[76] Krieger N. (1992). Overcoming the absence of socioeconomic data in medical records: validation and application of a census-based methodology. *American Journal of Public Health*.

[77] Shavers V. L. (2007). Measurement of socioeconomic status in health disparities research. *Journal of the National Medical Association*.

[78] Akinyemiju T. F., Soliman A. S., Johnson N. J. (2013). Individual and neighborhood socioeconomic status and healthcare resources in relation to black-white breast cancer survival disparities. *Journal of Cancer Epidemiology*.

[79] Kish J. K., Yu M., Percy-Laurry A., Altekruse S. F. (2014). Racial and ethnic disparities in cancer survival by neighborhood socioeconomic status in Surveillance, Epidemiology, and End Results (SEER) Registries. *JNCI Monographs*.

[80] Shariff-Marco S., Yang J., John E. M. (2014). Impact of neighborhood and individual socioeconomic status on survival after breast cancer varies by race/ethnicity: the neighborhood and breast cancer study. *Cancer Epidemiology Biomarkers and Prevention*.

[81] Ursin G., Bernstein L., Lord S. J. (2005). Reproductive factors and subtypes of breast cancer defined by hormone receptor and histology. *British Journal of Cancer*.

[82] Ma H., Bernstein L., Pike M. C., Ursin G. (2006). Reproductive factors and breast cancer risk according to joint estrogen and progesterone receptor status: a meta-analysis of epidemiological studies. *Breast Cancer Research*.

[83] Shinde S. S., Forman M. R., Kuerer H. M. (2010). Higher parity and shorter breastfeeding duration: association with triple-negative phenotype of breast cancer. *Cancer*.

[84] Yin D., Morris C., Allen M., Cress R., Bates J., Liu L. (2010). Does socioeconomic disparity in cancer incidence vary across racial/ethnic groups?. *Cancer Causes and Control*.

[85] Harris H. R., Willett W. C., Terry K. L., Michels K. B. (2011). Body fat distribution and risk of premenopausal breast cancer in the Nurses' Health Study II. *Journal of the National Cancer Institute*.

[86] Bernstein L., Lacey J. V. (2011). Receptors, associations, and risk factor differences by breast cancer subtypes: positive or negative?. *Journal of the National Cancer Institute*.

[87] Phipps A. I., Li C. I. (2014). Breastfeeding and triple-negative breast cancer: potential implications for racial/ethnic disparities. *Journal of the National Cancer Institute*.

[88] Sieri S., Chiodini P., Agnoli C. (2014). Dietary fat intake and development of specific breast cancer subtypes. *Journal of the National Cancer Institute*.

[89] Khankari N. K., Bradshaw P. T., Steck S. E. (2015). Dietary intake of fish, polyunsaturated fatty acids, and survival after breast cancer: a population-based follow-up study on Long Island, New York. *Cancer*.

